# Therapeutic Devices for Motor Symptoms in Parkinson’s Disease: Current Progress and a Systematic Review of Recent Randomized Controlled Trials

**DOI:** 10.3389/fnagi.2022.807909

**Published:** 2022-03-29

**Authors:** Joji Fujikawa, Ryoma Morigaki, Nobuaki Yamamoto, Teruo Oda, Hiroshi Nakanishi, Yuishin Izumi, Yasushi Takagi

**Affiliations:** ^1^Department of Advanced Brain Research, Institute of Biomedical Sciences, Graduate School of Medicine, Tokushima University, Tokushima, Japan; ^2^Department of Neurosurgery, Institute of Biomedical Sciences, Graduate School of Medicine, Tokushima University, Tokushima, Japan; ^3^Department of Neurology, Institute of Biomedical Sciences, Graduate School of Medicine, Tokushima University, Tokushima, Japan

**Keywords:** Parkinson’s disease, tremor, freezing of gait (FOG), gait, stimulation, invasive medical devices, non-invasive medical device

## Abstract

**Background:**

Pharmacotherapy is the first-line treatment option for Parkinson’s disease, and levodopa is considered the most effective drug for managing motor symptoms. However, side effects such as motor fluctuation and dyskinesia have been associated with levodopa treatment. For these conditions, alternative therapies, including invasive and non-invasive medical devices, may be helpful. This review sheds light on current progress in the development of devices to alleviate motor symptoms in Parkinson’s disease.

**Methods:**

We first conducted a narrative literature review to obtain an overview of current invasive and non-invasive medical devices and thereafter performed a systematic review of recent randomized controlled trials (RCTs) of these devices.

**Results:**

Our review revealed different characteristics of each device and their effectiveness for motor symptoms. Although invasive medical devices are usually highly effective, surgical procedures can be burdensome for patients and have serious side effects. In contrast, non-pharmacological/non-surgical devices have fewer complications. RCTs of non-invasive devices, especially non-invasive brain stimulation and mechanical peripheral stimulation devices, have proven effectiveness on motor symptoms. Nearly no non-invasive devices have yet received Food and Drug Administration certification or a CE mark.

**Conclusion:**

Invasive and non-invasive medical devices have unique characteristics, and several RCTs have been conducted for each device. Invasive devices are more effective, while non-invasive devices are less effective and have lower hurdles and risks. It is important to understand the characteristics of each device and capitalize on these.

## Introduction

Parkinson’s disease (PD) is a neurodegenerative disorder caused by the progressive loss of dopaminergic neurons in the midbrain (Lang and Lozano, 1998; Kalia and Lang, 2015). Its cardinal motor symptoms include tremor, rigidity, bradykinesia/akinesia, and postural instability (Dubois and Pillon, 1997). Pharmacotherapy is the mainstay treatment for patients with PD, and levodopa is the most effective drug for managing motor symptoms (Poewe and Mahlknecht, 2020). However, side effects such as dyskinesia and the on/off phenomenon have been associated with levodopa treatment (Holloway et al., 2004). Moreover, its effectiveness has decreased over the years (Obeso et al., 2017).

Invasive surgical alternatives to pharmacological treatments include stereotactic thalamotomy, deep brain stimulation (DBS), spinal cord stimulation (SCS), and continuous infusion of levodopa-carbidopa or apomorphine therapy. DBS is a widely used medical device therapy and surgical standard for patients with PD (Kleiner-Fisman et al., 2006). The use of SCS in patients with PD is promising for relieving concurrent pain conditions. The delivery of Duodopa^®^ intestinal gel directly into the small intestine reduces fluctuating motor symptoms and improves the quality of life (Olanow et al., 2014). However, the risk associated with the implantation of these invasive medical devices is a burden for patients with PD. For example, DBS may cause irreversible intracranial hemorrhages (Kleiner-Fisman et al., 2006).

**FIGURE 1:**
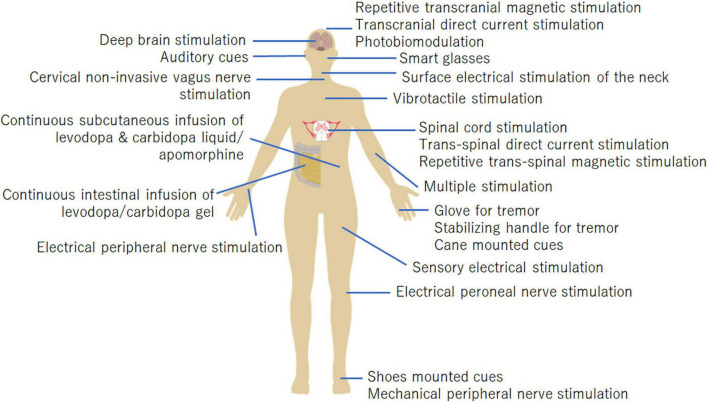
Body parts the medical devices are applied to. The stimulation targets of each device are shown in this figure.

Recently, alternative non-pharmacological/non-surgical approaches have been explored to alleviate PD symptoms. Non-invasive medical devices are mainly effective for improving tremors, freezing of gait (FOG), and gait. Emerging non-invasive medical devices primarily include management devices for tremors, non-invasive brain stimulation (NIBS), cueing devices for FOG, non-invasive vagus nerve stimulation (nVNS), vibrotactile stimulation devices, mechanical/electrical peripheral stimulation, and photobiomodulation devices. Each device adapts to different body parts (Figure 1) and has different properties and effects. In this study, we review emerging therapeutic devices for motor symptoms in PD.

## Methods

**FIGURE 2:**
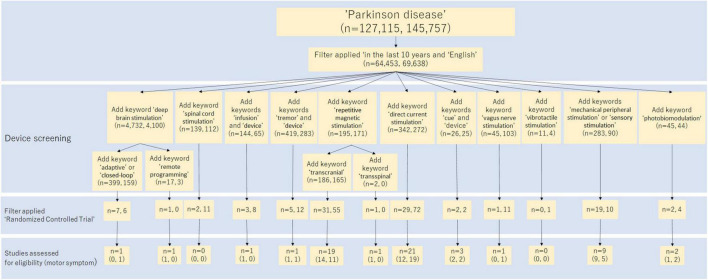
Flow diagram of the study selection process in the systematic review. This diagram shows how to search for studies in a systematic review. The numbers in parentheses show the breakdown of the number of search hits, with PubMed and Scopus listed in that order.

A narrative literature review and a systematic review of randomized control trials (RCTs) were conducted. These reviews summarized recent invasive and non-invasive medical devices and their effects on PD patients in the past 10 years. The search method is shown in Figure 2. All searches were performed on PubMed and Scopus. The study screening was done independently by two reviewers, JF and RM First, we searched for MeSH term “Parkinson’s disease.” English language literature in the past 10 years was reviewed. Next, we added the device name to the search terms and performed a search for each device, as shown in Figure 2. Articles found in this search were screened based on title and abstract, then based on the full text. In the narrative literature review, we selected literature related to motor symptoms. For the systematic review, RCTs were extracted out from the search results. We manually reviewed these papers and selected the ones that fit. The RCT studies selected from the search are shown in Table 1.

**TABLE 1:** Randomized controlled trials of medical devices.

Device/Method	Invasive/Non-invasive	CE marking and FDA certification for PD	References	Subject	Efficacy
Adaptive DBS	Invasive	CE marking and FDA approved	Sasaki et al., 2021	12 PD patients	Both programed using a standard of care and the closed-loop algorithm improved UPDRS Part III scores and sensor-based predominantly, but there was no significant difference between the two methods. Median UPDRS Part III was 37.5 points at baseline, 22.0 points for programed using a standard of care and 23.5 points (20.3–27.0) for a closed-loop algorithm. The programming steps were significantly reduced in the closed-loop compared to the existing method.

DBS with remote programming	Invasive	CE marking and FDA approved	Li et al., 2017	64 PD patients	Bilateral wireless programming STN -DBS significant decrease in the UPDRS motor scores were observed for the test group in the off-medication state (25.08 ± 1.00) vs. the control group (4.20 ± 1.99).

LCIG	Invasive	CE marking and FDA approved	Olanow et al., 2014	66 PD patients	LCIG significantly reduced “Off” time by a mean (± SE) of 1.91 ± 0.57 h (*P* = 0.0015) and increased “On” time without troublesome dyskinesia by a mean of 1.86 ± 0.65 h (*P* = 0.006).

Tremor’s glove	Non-invasive	Not approved	Jitkritsadakul et al., 2017	30 PD patients	During stimulation, significant reduction in RMS angular velocity (as percentage) in every axis and peak magnitude in axis (x-, y-) and UPDRS tremor score (glove : 5.27 ± 2.19, sham : 4.93 ± 2.37) were found with Tremor’s glove compared to the sham groups (*p* < 0.05, each).

rTMS over M1	Non-invasive	Not approved	Benninger et al., 2012	26 PD patients	50 Hz rTMS did not improve gait, bradykinesia, global and motor UPDRS.
			Maruo et al., 2013	21 PD patients	rTMS over M1 significantly improved UPDRS Part III, visual analog scale, the walking test, self-assessment motor score, and finger tapping measurement. No significant improvement was observed in depression and apathy scales.
			Yang et al., 2013	20 PD patients	Effectiveness study of combination of rTMS and treadmill training. Significant time effects on almost all corticomotor and functional variables and it suggested combination of rTMS and treadmill training improve walking performance.
			Kim et al., 2015	17 PD patients	The TUG and UPDRS Part III showed significant ameliorations over time.
			Chang et al., 2016	8 patients with atypical parkinsonism	FOGQ, turn steps, TUG task and UPDRS Part III revealed significant improvements.
			Cohen et al., 2018	42 PD patients	A study of repetitive deep transcranial magnetic stimulation using H5 coils. Although repetitive deep transcranial magnetic stimulation treatment exhibited some motor improvements, we could not demonstrate an advantage for real treatment over sham.
			Khedr et al., 2019	52 PD patients	Comparing the effects of 20 and 1 Hz. Both improve PD motor function, but 20 Hz rTMS is more effective.

rTMS over SMA	Non-invasive	Not approved	Chung et al., 2020	51 PD patients	The 1 and 25 Hz rTMS groups produced a greater improvement in fastest walking speed at 1 day and 3 months postintervention than the sham group.
			Shirota et al., 2013	106 PD patients	At week 20, 1 Hz stimulation showed an improvement of 6.84 points on the UPDRS Part III.
			Sayin et al., 2014	17 PD patients	1 Hz rTMS reduced levodopa-induced dyskinesias lasting 24 h without altering motor performance
			Ma et al., 2019	28 PD patients with FOG	Beneficial effects on FOG and some gait parameters, but no improvement in sequence effects.
			Mi et al., 2019	30 PD patients with FOG	Significantly decreased FOGQ (up to -2.13 points, 95% CI -2.97 to -1.29). Significant improvements of UPDRS Part III (up to -6.69, 95%CI -8.73 to -4.66) and gait variables.
			Ji et al., 2021	42 PD patients	UPDRS Part III score significant decrease in the rTMS group (from 28.0 ± 2.12 at baseline to 20.6 ± 1.82 at Week 2; *p* < 0.0001).

rTMS over DLPFC	Non-invasive	Not approved	Li et al., 2015	132 PD patients	Comparison of the effects of istradefylline and rTMS. There was no significant difference in the UPDRS Part III score, and istradefylline and rTMS had comparable efficacy and tolerability.
			Zhuang et al., 2020	33 PD patients	Compared to baseline, active rTMS showed significant improvement in the UPDRS Part III and Non-motor Symptom Questionnaire at 1 month, and the change in scores persisted for 3 months after rTMS intervention.

rTMS over M1, SMA, DLPFC	Non-invasive	Not approved	Lee et al., 2014	20 patients with parkinsonism	tDCS over M1 and DLPFC were significant improvements in TUG test times and UPDRS Part III scores.
			Yokoe et al., 2018	19 PD patients	rTMS over M1 or SMA was able to significantly improve motor symptoms, but it could not clearly improve mood disorders.

rTMS over M1, DLPFC	Non-invasive	Not approved	Brys et al., 2016	50 PD patients	tDCS over M1 was able to significantly improve motor function; there was no benefit from combining M1 and DLPFC stimulation.

rTMS over M1, PFC	Non-invasive	Not approved	Spagnolo et al., 2020	59 PD patients	rTMS (M1-PFC and M1 combined) significantly greater improvement compared to sham in UPDRS Part III total score (*p* = 0.007), tremor subscore (*p* = 0.011), and lateralized sub-scores (*p* = 0.042 for the more affected side; *p* = 0.012 for the less affected side).

Multitarget tDCS (M1 and left DLPFC)	Non-invasive	Not approved	Dagan et al., 2018	20 PD patients with FOG	Significant improvements of TUG, and the Stroop test.
			Manor et al., 2021	77 PD patients with FOG	Decreased self-reported FOG severity and increased daily living step counts. However, demonstrated no advantage for tDCS in laboratory-based FOG-provoking test.

tDCS over M1	Non-invasive	Not approved	Valentino et al., 2014	10 PD patients	Reduction in number and duration of freezing of gait episodes, significant improvements of UPDRS Part III.
			Cosentino et al., 2017	14 PD patients	tDCS induced significant changes in cortical excitability and motor performances of both hands significantly improved.
			Broeder et al., 2019	10 PD patients and 10 healthy control subjects	Funnel task on a touch-sensitive tablet was found significant reduction in upper limb freezing episodes.

tDCS over DLPFC	Non-invasive	Not approved	Beretta et al., 2020b	24 PD patients	tDCS over M1 improved the postural response to external perturbation in PD, with better response observed for 2 mA compared with 1 mA.
			Swank et al., 2016	10 PD patients	Participants performed TUG single and dual task conditions. It did not significantly improve gait.
			Lattari et al., 2017	17 PD patients	Investigate the impact on functional mobility and balance. Significant improvement in Berg Balance Scale, Dynamic Gait Index, TUG.
			Bueno et al., 2019	20 PD patients	Statistically significant differences were found for Trail Making Test part B in active and sham groups. For the Verbal Fluency test differences were found only within the group that received real stimulation.
			Mishra and Thrasher, 2021	20 PD patients	In the dual-task condition, participants walked faster at 15 min (*p* = 0.017) and 30 min (*p* < 0.01) after anodal tDCS ceased compared to sham. Similarly, participants generated a higher number of words per minute at 15 min (*p* = 0.017), and 30 min (*p* < 0.01) after anodal tDCS ceased compared to sham.

tDCS with physical training	Non-invasive	Not approved	Kaski et al., 2014b	16 PD patients	tDCS with physical training increased gait velocity (mean = 29.5%, *SD* = 13; *p* < 0.01) and improved balance (pull test: mean = 50.9%, *SD* = 37; *p* = 0.01) compared with tDCS alone.
			Schabrun et al., 2016	16 PD patients	Gait speed, step length and cadence improved in the active and sham groups, under all dual-task conditions. This effect was maintained at follow-up. There was no difference between the active and sham tDCS groups.
			Costa-Ribeiro et al., 2017	22 PD patients	tDCS + cueing gait training group and sham tDCS + cueing gait training group demonstrated similar gains in all outcome measures, except for the stride length. The number of participants who showed minimal clinically important differences was similar between groups.
			Fernandez-Lago et al., 2017	18 PD patients	Evaluated the combination of treadmill walking and tDCS over M1. It improved walking performance and modulated spinal and corticospinal parameters in a similar way.
			Yotnuengnit et al., 2018	53 PD patients	After intervention, group 1 (only tDCS) demonstrated a significant increase in gait speed by 0.13–0.14 m/s (17.8–19.2%) and an increase in step length by 5.9–6.1 cm (14.0–14.5%), whereas group 2 (tDCS and physical therapy) revealed a significant increase in gait speed by 0.10–0.13 m/s (14.9–19.4%) and step length by 4.5–5.4 cm (10.6–12.8%) and group 3 (sham tDCS and physical therapy) showed a significant increase in gait speed by 0.09–0.14 m/s (13.0–20.3%) and step length by 3.0–5.4 cm (6.8–12.3%).
			Horiba et al., 2019	18 PD patients	Mirror visual feedback combined with tDCS over M1. Apply tDCS, the number of ball rotations in accordance with input-output function at 150% intensity was significantly increased after day 1 and retained until day 2.
			Conceicao et al., 2021	17 PD patients	Investigated the effects of tDCS over the prefrontal cortex with cycling. Participants decreased step time variability (effect size: -0.4), shortened simple and choice reaction times (effect sizes: -0.73 and -0.57, respectively), and increased PFC activity.
			Lee and Kim, 2021	30 PD patients	tDCS combined with visual cueing training. Results showed a significant decrease in UPDRS Part III score and a significant increase in functional gait assessment and cadence.

tDCS over SMA, M1	Non-invasive	Not approved	da Silva et al., 2018	17 PD patients	Significant group difference with gait cadence (P = .014, *d* = 0.87), indicating its reduction after tDCS (-0.28 [-1.16, 0.01] steps/s) compared with sham tDCS group (0.17 [0.00, 0.40] steps/s).

tDCS over primary and premotor cortices	Non-invasive	Not approved	Kaski et al., 2014a	1 PD patient	Evaluated the combination of tango dance and tDCS. Significant improvements of trunk velocity and TUG.

tDCS of the cerebellum	Non-invasive	Not approved	Lima de Albuquerque et al., 2020	22 PD patient	Evaluated motor performance by a visuomotor isometric precision grip task and a rapid arm movement task. From results indicate that an acute application of tDCS of the cerebellum does not enhance motor performance in hand and arm tasks in PD.

tDCS over the left sensorimotor (anode) and right frontal areas (cathode)	Non-invasive	Not approved	Schoellmann et al., 2019	10 PD patients and 10 healthy control subjects	Motor UPDRS Part III hemibody score of the right upper extremity (items 22–25) improved. Neurophysiological features indicated a motor-task-specific modulation of activity and coherence from 22 to 27 Hz after tDCS.

rTSMS	Non-invasive	Not approved	Arii et al., 2014	37 PD patients with camptocormia	Evaluated immediate effect of rTSMS on camptocormia. The flexion angle in the standing position significantly decreased by a mean of 10.9° and flexion angle while sitting significantly decreased by 8.1°.

Cueing by smart glass (Google Glass)	Non-invasive	Not approved	Zhao et al., 2016	12 PD patients with FOG	Participants were overall positive about the usability of the Google Glass. However, freezing of gait did not significantly decrease.

Laser light visual cueing	Non-invasive	Not approved	Bunting-Perry et al., 2013	22 PD patients with FOG	The laser beam applied as a visual cue. However, it did not diminish freezing of gait.

Visual cues combined with treadmill training	Non-invasive	Not approved	Schlick et al., 2016	23 PD patients	Combining visual cues with treadmill training significantly improved TUG, gait speed, and stride length as compared to not combining the two.

nVNS	Non-invasive	Not approved	Mondal et al., 2021	33 PD patients with FOG	The velocity increased by 16% (*p* = 0.018), step length increased by 11% (*p* = 0.021), and step time reduced by 16% (*p* = 0.003) in the active nVNS group.

Mechanical peripheral stimulation	Non-invasive	CE marking and FDA approved (Gondola)	Barbic et al., 2014	16 PD patients	Mechanical stimulation of the feet increases stride length and gait speed, increases upright rotation speed, and decreases step count.
			Quattrocchi et al., 2015	11 PD patients	Automatic mechanical peripheral stimulation was found to increase resting-state functional connectivity in the sensorimotor cortex, striatum, and cerebellum.
			Galli et al., 2018	28 PD patients and 32 healthy control subjects	Mean velocity, stride length, ankle ROM, and knee ROM significantly improved (*P* < 0.05) after the sessions compared with pre-sessions only in the intervened group.
			Kleiner et al., 2018	30 PD patients	Significant for gait asymmetry [*F*(3, 204) = 7.420; *P* = 0.0001], mean step length [*F*(3, 201) = 3.570; *P* = 0.015], step length coefficient of variation [*F*(3, 207) = 7.093; *P* = 0.0001], step time variability standard deviation [*F*(3, 210) = 3.223; *P* = 0.024], step time variability coefficient of variation [*F*(3, 210) = 5.503; *P* = 0.001], and gait velocity [*F*(3, 210) = 5.070; *P* = 0.027].
			Pagnussat et al., 2018	33 PD patients	Effective stimulation group showed significantly higher serum levels of brain-derived neurotrophic factor and lower serum levels of cortisol compared to sham stimulation group. Gait velocity, stride length, and TUG performance were significantly improved in effective stimulation group.
			Pinto et al., 2018	30 PD patients	Significant improvement in hip internal-external rotation between intervened and sham-control group (*P* = 0.018). Hip internal-external rotation, stride length, step length, and gait speed were significantly improved (*P* ∼ 0.000) after eight sessions compared to pre-conditions.
			Prusch et al., 2018	33 PD patients	No positive effects on center of pressure parameters (no positive effect in improving static postural control).
			Pagnussat et al., 2020	25 PD patients	There were no changes in brain activity by task-based fMRI. Resting-state fMRI showed increase in brain connectivity in areas related to sensory processing and sensorimotor integration.

Surface electrical stimulation of the neck (submental region)	Non-invasive	Not approved	Baijens et al., 2013	90 PD patients with dysphagic	No statistically significant differences in fiber optic endoscopic evaluation of swallowing and videofluoroscopy of swallowing outcome variables were found.

Photobiomodulation	Non-invasive	Not approved	Santos et al., 2019	35 PD patients	Significantly improved the walking speed in the fast rhythm of the 10 m walking test by an average of 0.33 m/s.
			Liebert et al., 2021	12 PD patients	Measures of mobility, cognition, dynamic balance, and fine motor skill were significantly improved (*p* < 0.05) with photobiomodulation treatment for 12 weeks and up to 1 year.

*DBS, deep brain stimulation; DLPFC, Dorsolateral prefrontal cortex; FDA, Food and Drug Administration; fMRI, functional magnetic resonance imaging; FOG, Freezing of Gait; FOG-Q, Freezing of Gait Questionnaire score; LCIG, continuous infusion of levodopa-carbidopa gel; M1, Primary motor cortex; nVNS, non-invasive vagus nerve stimulation; PD, Parkinson’s disease; PFC, pre-frontal cortices; RCT, randomized controlled trial; RMS, the root mean square; ROM, range of motion; rTMS, repetitive transcranial magnetic stimulation; rTSMS, repetitive trans-spinal magnetic stimulation; SMA, supplementary motor area; STN, subthalamic nucleus; tDCS, transcranial direct current stimulation; TUG, timed up and go; UPDRS, the Unified Parkinson’s Disease Rating Scale.*

## Results

### Narrative Literature Review

#### Deep Brain Stimulation

DBS is the surgical standard for patients with movement disorders such as PD, essential tremor (ET), and dystonia. DBS is a highly effective and widely used treatment for PD patients and is probably the most important advance in PD treatment since the introduction of levodopa (Kleiner-Fisman et al., 2006; Benabid et al., 2009; Bronstein et al., 2011). The target of stimulation is either the subthalamic nucleus (STN) or internal globus pallidus (GPi) (Sako et al., 2014). The ventral intermediate nucleus of the thalamus (VIM) is also used to improve tremors (Morigaki et al., 2011a,b). Recently, advanced imaging sequences have enabled direct visualization of anatomical targets, and patient-specific DBS has been performed (Neudorfer et al., 2022). DBS has been demonstrated to improve motor function including FOG (Krack et al., 2003; Huang et al., 2018), camptocormia (Sako et al., 2009; Chan et al., 2018), tremor (Morigaki et al., 2011a,b), and cognitive dysfunction. Although DBS has a lower complication rate than stereotactic thalamotomy (Tasker, 1998; Pahwa et al., 2001), intracranial hemorrhage and infection still occur in 3.9 and 1.6% of the patients, respectively (Kleiner-Fisman et al., 2006). There is also a risk of secondary psychiatric effects (Piasecki and Jefferson, 2004; Kinoshita et al., 2018). Long-term effects have been shown to last for more than 10 years for tremors. In many cases, activity of daily living can be maintained for a long time, and patient satisfaction remains high even after 10 years of follow-up (Hitti et al., 2019). However, since DBS is not effective for all patients, it is necessary to determine if the patient is an appropriate candidate. It is also important to confirm the diagnosis of idiopathic PD. A thorough neurological examination is necessary because some neurological disorders can mimic the signs and symptoms of idiopathic PD (Machado et al., 2006). Also, DBS effects for gait impairment, postural instability, postural abnormalities, FOG, and other axial motor signs are under debate (Fasano et al., 2015). Expert opinion showed that the risk/benefit ratio in the elderly for STN DBS is not very favorable; unlike STN DBS, there is no clinical indication that the outcome of VIM DBS is in any way affected by age. It must be determined on an individual basis, considering the need for treatment, risk factors for complications, and general life expectancy (Lang et al., 2006). The precise underlying mechanisms of DBS remain a matter of debate. A leading hypothesis is that DBS restores aberrant neuronal firing to non-pathological rates by applying high-frequency trains of electrical stimulation (Muller and Robinson, 2018). DBS devices are also increasing in functionality as research advances, such as a variety of stimulation methods [interleaved stimulation (Wojtecki et al., 2011; Baumann et al., 2012; Aquino et al., 2019), dual-target

stimulation (Hollingworth et al., 2017), directional stimulation (Schupbach et al., 2017), current steering (Barbe et al., 2014)], and support for magnetic resonance imaging (Larson et al., 2008; McElcheran et al., 2019). Studies have also been conducted to investigate the corresponding effects on stimulus frequency (Karl et al., 2020). Stimulation parameters may change the effects and long-term consequences. In this section, we introduce two emerging technologies in DBS: adaptive DBS (aDBS) and remote programming for DBS adjustment, both of which have seen significant growth and attention in recent years.

##### Adaptive Deep Brain Stimulation

DBS devices that operate on the principle of closed-loop interaction are called aDBS. A closed-loop system can sense the effect of stimulation and adjust the stimulation in response to the observed effect. Recently, a closed-loop stimulation technology based on local field potentials (LFPs) in target structures as biomarkers has been used for the treatment of PD subjects (Marceglia et al., 2009; Giannicola et al., 2010; Little et al., 2013; Sasaki et al., 2021). Compared to the cortical neural signals such as electroencephalography (EEG), electrocorticography, and magnetoencephalography, LFP signals from deep electrodes provide direct changes in basal ganglia function (Einevoll et al., 2013). In PD, increased synchrony of neuronal networks in the beta band (13–35 Hz) is associated with reduced segregation of parallel processes, leading to reduced specificity of motor programs (Mink, 1996; Pessiglione et al., 2005). The amplitude of spectral peaks in the beta band correlates with the severity of symptoms (Neumann et al., 2016, 2017) and is reduced by dopaminergic medication and DBS (Kuhn et al., 2009). Beta-power is an ideal candidate for aDBS as a biomarker (Marceglia et al., 2009; Giannicola et al., 2010; Little et al., 2013) and aDBS using LFPs has been confirmed to have advantages over conventional DBS (cDBS). aDBS reportedly prevents several complications related to cDBS, including gait and speech disturbances, and was appropriately modulated by levodopa administration, with a further reduction in stimulation, potentially preventing excessive combined therapy and dyskinesias (Little et al., 2016a,b). Bocci et al. (2021) reported that the Unified PD Rating Scale (UPDRS) part III scores, the Rush scale for dyskinesias, and the total electrical energy delivered to the tissues per second (TEEDs) were significantly lower in the aDBS session during an 8-h stimulation protocol, in conjunction with chronic levodopa assumption and without restriction on patients’ activities. The safety and effectiveness of aDBS stimulation compared to cDBS in a daily session in terms of motor performance and TEEDs in patients with PD are shown. Little et al. (2013) compared the effectiveness of aDBS with that of cDBS. aDBS showed 66% (unblinded) and 50% (blinded) improvements in motor scores, which were 29% (unblind) and 27% (blinded) significantly better than cDBS. These improvements were achieved with a 56% reduction in stimulation time and a significant reduction in energy requirements compared to cDBS.

In the future, a closed-loop system that uses neurotransmitters such as dopamine, histamine, adenosine, serotonin, and glutamate release as a control variable has been proposed (Griessenauer et al., 2010; Shon et al., 2010; Chang et al., 2012a,b; Chang et al., 2013). A 30-minute baseline of dopamine during a DBS surgery was quantified using microdialysis (Kilpatrick et al., 2010). Kishida et al. (2011, 2016) performed the voltammetric measurement of real-time dopamine dynamics in patients with PD using fast scan cyclovoltammetry (FSCV). One study targeted another neurotransmitter, adenosine. Real-time in vivo FSCV neurochemical recordings in patients with tremors showed that DBS induces adenosine release concurrent with tremor arrest (Chang et al., 2012b). It has become clear that although clinical monitoring of neurotransmitters is technically feasible, and these sensing technologies need to be improved to measure dopamine over long periods (days, months, years). This will require the development of technologies that can solve current problems such as biofouling and material degradation to enable reliable long-term recording. Furthermore, the improvement of current biosensors or the development of new biosensors to accommodate chronic implantation and the method for information processing and decision making are also essential.

##### Remote Programming for Deep Brain Stimulation Adjustment

Remote DBS programming has been implemented in clinical practice (Li et al., 2017; Zhang et al., 2020; Ma et al., 2021; Xu et al., 2021). Unfortunately, many patients do not achieve the expected DBS outcome due to inadequate or suboptimal programming (Farris and Giroux, 2013). Optimization of DBS parameters is usually attained within three to 6 months, during four to five programming sessions (Bronstein et al., 2011). Additionally, the parameters need to be adjusted over the long term to maintain this effect. The physician responsible for postoperative programming should be an expert in both DBS and medical management of PD patients, but patients living in remote areas may have inadequate access and thus may not receive adequate adjustments. Thus, teleprogramming has received increasing attention in recent years, partly because of the coronavirus disease 2019 pandemic. Ma et al. (2021) followed 90 patients for a total of 386 remote programming visits over 27 months. Their questionnaire survey demonstrated that each remote programming visit saved ≥ 2000 Chinese yuan for 76.7% of the patients and ≥ 12 h for 90.0% of the patients, compared with the on-site programming visit. Respondents also rated the acceptability of the remote programming platform highly. Moreover, 89% of the patients were satisfied with the remote programming. Significant improvement in UPDRS Part III was also achieved in another study of 32 patients conducted by Xu et al. (2021). LeMoyne et al. (2019a, b, 2020a, 2020b, 2020c) quantified the tremor of PD patients via a wireless inertial sensor system with connectivity to Cloud computing resources attached to the dorsal side of the hand and successfully classified the stimulus amplitude of DBS. Machine learning was used for classification, with neural networks, J48 decision trees, K-nearest neighbor, support vector machine, logistic regression, and random forest, achieving 100% accuracy in certain conditions. An online system for handling such objective and quantitative data is essential for remote DBS coordination.

#### Spinal Cord Stimulation

The use of SCS in patients with PD is promising for relieving concurrent pain conditions (Fenelon et al., 2012; Cai et al., 2020). Notably, SCS has recently also been shown to be effective for locomotive symptoms in patients with PD (Hassan et al., 2013); therefore, SCS might be a viable alternative therapy to DBS for the management of PD symptoms (Pinto de Souza et al., 2017). Santana et al. (2014) showed that SCS at the upper thoracic level in a primate model of PD improved freezing, hypokinesia, postural instability, and bradykinesia, which is associated with a reduction in beta-frequency oscillation within the cortico-basal ganglia circuitry.

In human case reports, high cervical SCS (C2-C3) yielded incongruent results (Thevathasan et al., 2010; Hassan et al., 2013). Cervical SCS with tonic waveform requires a long latency (more than 3 months) for motor improvement (Mazzone et al., 2019). Contrastingly, the improvement by cervical SCS with burst waveform stimulation showed acute and larger improvements in motor symptoms, including tremor, gait parameters, and pain (Mazzone et al., 2019). Similarly, thoracic SCS (T6-T12) reduced gait impairment, rigidity, abnormal posture, and tremor (Agari and Date, 2012; Fenelon et al., 2012; Landi et al., 2013; Nishioka and Nakajima, 2015; Samotus et al., 2018; Hubsch et al., 2019). Thoracic SCS with burst waveform stimulation also improves pain, gait, and stooping posture (Kobayashi et al., 2018; Furusawa et al., 2020). Some authors have advocated SCS as a possible salvage therapy for DBS with decreased efficacy over the years (Agari and Date, 2012; Landi et al., 2013; Akiyama et al., 2017; Pinto de Souza et al., 2017). However, a 1-year single prospective open-label pilot study revealed no beneficial effect of thoracic SCS in six pain-free advanced PD patients with significant axial symptoms (Prasad et al., 2020). The possibility of quick habituation for tonic stimulation is advocated for the gradual loss of clinical benefit in axial symptoms over time and the ongoing pilot trial of cyclic stimulation (Cury et al., 2020).

The major complications of SCS include infection, lead migration or breakage, subcutaneous hematoma, and discomfort due to the pulse generator (Nissen et al., 2019). The infection rate is 3–6% (Nissen et al., 2019); however, the majority of SCS studies in patients with PD were either uncontrolled or controlled trials with small sample sizes. Thus, well-designed clinical trials, including double-blind and placebo-controlled arms with large sample sizes and specific stimulation protocols are warranted to generate solid evidence regarding the effectiveness of SCS (Fonoff et al., 2019; Rahimpour et al., 2021).

#### Continuous Infusion of Levodopa-Carbidopa or Apomorphine Therapy

PD patients are mainly treated with oral administration of levodopa and dopamine agonists during the early stage (Fahn and Sulzer, 2004). However, the duration of the response to treatment becomes shorter, and side effects—such as hallucinations—are marked as the progression of the disease stage. Complications such as fluctuations, including the wearing-off phenomenon and dyskinesia, can be seen in patients who have been receiving long-term oral levodopa (Eggert et al., 2008). This fluctuation of symptoms might be caused by the short half-life of levodopa (Nyholm et al., 2005). Continuous drug delivery, allowing for continuous dopaminergic stimulation, is necessary for patients with advanced-stage PD (van Laar, 2003; Nyholm et al., 2005; Gershanik and Jenner, 2012). Dopaminergic stimulation can be prolonged by two methods: the simultaneous administration of enzyme inhibitors to prevent the metabolism of levodopa and continuous administration of dopaminergic agents, not orally. Continuous levodopa administration was reported to be effective for the reduction of dyskinesia and off-time by stabilizing plasma levodopa/carbidopa levels and providing continuous stimulation of dopaminergic receptors in the striatum (Sage et al., 1988; Politis et al., 2017).

The methods currently available for continuous infusion are subcutaneous apomorphine, levodopa/carbidopa liquid, and enteral levodopa/carbidopa gel (Duodopa^®^, Abbott, Allschwil, Switzerland). Continuous subcutaneous apomorphine infusion is effective on motor function and alleviates motor fluctuations in patients with PD and, it can also significantly reduce off-time (Garcia Ruiz et al., 2008). Various types of apomorphine infusion approaches such as inhale, patch pump, and sublingual administrations have been developed (Titova and Chaudhuri, 2016). In recent years, the efficacy of these approaches on motor function has been assessed (Olanow et al., 2020) and sublingual apomorphine has received Food and Drug Administration (FDA) approval (Larson and Simuni, 2022). Duodopa^®^ can be delivered to the jejunum via a percutaneous gastrojejunostomy tube, which is connected to a portable infusion pump filled with Duodopa^®^. Duodopa^®^ intestinal gel can shorten off-time while extending on-time without dyskinesia (Olanow et al., 2014). Furthermore, Slevin et al. (2015) and Merola et al. (2016) investigated on-time with/without troublesome dyskinesia with Duodopa^®^ treatment. Although there was no significant difference in on-time with troublesome dyskinesia between the control and Duodopa^®^ groups, significant differences were observed in on-time without troublesome dyskinesia and off-time, suggesting that Duodopa^®^ had a positive effect. However, the pooled results showed that Duodopa^®^ intestinal infusion did not significantly improve the UPDRS total score, including parts II and III (Wang et al., 2018). Contrastingly, a cost-utility analysis was performed in the United Kingdom and showed that Duodopa^®^ intestinal infusion increased quality-adjusted life years while also being cost-effective. Thus, it seems that Duodopa^®^ is effective in reducing fluctuating motor symptoms and improving quality of life, and so might be a useful tool to treat patients with advanced-stage PD. The most frequent adverse events associated with Duodopa^®^ were surgery-associated adverse events, such as infection and inflammation, which may be associated with morbidity and mortality in some patients. Tube obstruction, dislocation of the catheter tip, pump failure, and pull-out of the tube were reported to be related to the infusion system. Furthermore, drug-induced events, such as hallucinations, dystonia, worsened dyskinesia, acute peripheral neuropathy, psychosis, weight loss, and homocysteine concentrations have also been reported (Nyholm, 2012).

In recent years, another method for continuous levodopa administration is the continuous subcutaneous infusion of levodopa using ND0612 (NeuroDerm, Rehovot, Israel) (Olanow et al., 2021). ND0612 is a drug/device combination consisting of a subcutaneous pump and a liquid formulation. This formulation contains excipients to allow for low infusion rates. ND0612 administrates a liquid levodopa/carbidopa continuously, that stabilizes plasma levodopa levels compared to standard oral levodopa (Giladi et al., 2021). Olanow et al. (2021) evaluated the potential benefits of a 14-h (waking day) infusion compared to a 24-h infusion. The off-time for the overall population was significantly reduced by 2.0 h compared to baseline. On-time with no troublesome dyskinesia was significantly increased from baseline by 3.3 h, and on-time with moderate/severe dyskinesia was also significantly reduced by 1.2 h. The reduction in off-time was 1.5 h larger in the 24-h group than in the 14-h group. Notably, complete resolution of off-time was observed in 42% of patients in the 24-h group. Therefore, subcutaneous administration might both avoid fluctuations in absorption resulting from delayed gastric emptying and reduce surgical risks compared to Duodopa^®^ (Nilsson et al., 1998; Nyholm and Lennernas, 2008). Poewe et al. (2021) evaluated the 1-year safety data of ND0612. After evaluation by 214 patients, most patients experienced infusion site reactions such as particularly nodules (30.8%) and hematomas (25.2%). However, most cases were mild to moderate, and only 10.3% of the patient discontinued treatment. This indicated that ND0612 is generally safe.

Continuous subcutaneous infusion of apomorphine has been developed by Mitsubishi Tanabe/Neuroderm as ND0701. ND0701 is a novel concentrated apomorphine formulation for apomorphine-based continuous subcutaneous infusion. A study by Ramot et al. (2018) and Larson and Simuni (2022) showed that it was safe and well-tolerated and had similar bioavailability to commercially available apomorphine formulations.

#### Non-invasive Devices for Tremor

Tremor is the most common movement disorder and is defined as a rhythmic, involuntary oscillating movement (Bhatia et al., 2018). ET is the most common tremor worldwide. Although ET affects any part of the body, this tremor occurs most often in the hands, especially at the time of action—such as drinking and writing. Contrastingly, approximately 70% of PD patients also experience a tremor during the course of illness (Martin et al., 1973; Hughes et al., 1993; Pal et al., 2002), although this resting tremor tends to be a pill-rolling tremor, which looks like holding a pill between the thumb and forefinger and rolling it around. For patients with PD, tremor is one of the most disabling symptoms, in addition to slowed movement, rigid muscles, and postural balance impairment. Although resting tremor is a well-known symptom of PD, postural tremor associated with PD can cause more disability than typical resting tremors (Lance et al., 1963; Koller et al., 1989). Furthermore, tremor can be associated with a certain degree of shame in social situations among PD patients. Thus, the management of tremors may be important for patients with PD to improve their activities of daily living.

##### Active Devices

Pharmacological treatments can suppress tremors; for instance, levodopa and propranolol are used to alleviate resting tremors; primidone and propranolol for hand postural tremors; and beta-blockers, anticholinergic drugs, and primidone for kinetic tremor (Zesiewicz et al., 2011; Shanker, 2019; Poewe and Mahlknecht, 2020). However, although medication may be useful for suppressing tremors, it may incur mental or physical side effects such as addiction, hypotension, decrement of heart rate, and a feeling of thirst. Approximately 30% of patients with tremors do not respond to pharmacological treatment or experience intolerable secondary effects (Koller and Vetere-Overfield, 1989). Moreover, up to 56% of patients eventually discontinue their medication because of these secondary effects or a lack of efficacy (Diaz and Louis, 2010). For patients with tremors unresponsive to medication, stereotactic surgery—such as DBS, gamma knife radiosurgery, and focused ultrasound—can be a viable alternative. However, considering the severe side effects and complications caused by both medication and surgical intervention, non-surgical device treatments for reducing tremors may represent a good solution.

Active devices transfer external force or energy from devices into nerves and/or muscles to reduce tremors (Table 2). These devices also measure tremor characteristics using a gyroscope, accelerometer, and electromyogram. Peripheral nerve stimulation has been reported to be useful in reducing tremors—especially in patients with ET (Kim et al., 2020; Yu et al., 2020; Reis et al., 2021)—and is thought to evoke central activity via VIM, a thalamic target widely accepted to improve tremor with DBS (Morigaki et al., 2011a,b; Morigaki and Goto, 2015). Lin et al. (2018) reported the effect of median and radial nerve stimulation using a wrist band in patients with PD. They compared the tremor research group’s ET rating assessment scale and spiral drawing tasks pre- and post-stimulation and the scores decreased significantly after stimulation.

**TABLE 2:** Summary of non-invasive devices for patients with tremor.

Device/Method	CE marking and FDA certification	References	Subject	Efficacy	Adverse event
Cala ONE	FDA-approved Class II medical device	Beringhause et al., 1989	77 patients with ET	Improvement of upper limb TETRAS tremor score and subject related ADL score	Irritation, discomfort, burns
Cala TRIO™	FDA-registered Class I medical device	Hendriks et al., 1991	205 patients with ET	Improvement of upper limb TETRAS tremor score and subject related ADL score	Irritation, discomfort, burns
MOTIMOVE	CE marking approved	Popovic Maneski et al., 2011	3 patients with ET and	67% tremor suppression	Muscles fatigue
			4 patients with PD		
Tremor’s Glove	Not approved	Jitkritsadakul et al., 2017	30 patients with PD	Reduced UPDRS score	Muscles fatigue
Tremor Neurorobot	Not approved	Gallego et al., 2013	4 patients with ET and	52% tremor suppression	Muscles fatigue
			2 patients with PD		
Vib-Bracet	Not approved	Buki et al., 2018	1 patient with PD	85% Tremor suppression	Not reported
Liftware Steady™	FDA approved	Sabari et al., 2019	15 patients with ET	Improvement of the Fahn-Tolosa-Marin Tremor Rating Scale during eating, and transferring objects	Not reported
				73% tremor suppression	

*ADL, activity of daily living; ET, essential tremor; FDA, Food and Drug Administration; PD, Parkinson’s disease; TETRAS, the Tremor Research Group’s Essential Tremor Rating Assessment Scale; UPDRS, the Unified Parkinson’s Disease Rating Scale.*

The Cala One device (Cala Health, CA, United States) was the first wearable electrical nerve stimulator approved by the FDA (Beringhause et al., 1989). The clinical trial using the new version of the device, Cala TRIO™ (Cala Health, CA, United States), was completed in 2019 (Hendriks et al., 1991). An accelerometer built into this device can assess the frequency of tremors, which enables the individualized calibration of the stimulation intensity. The Cala TRIO™ includes two working electrodes positioned over the median and radial nerves deliver electrical signals that intermittently excite these nerves, while the VIM is stimulated through peripheral sensory nerves of the median and radial nerves, similar to DBS (Bathien et al., 1980; Hanajima et al., 2004a,b). A study with five patients with tremors due to either ET or PD demonstrated its efficacy for tremor suppression (Dosen et al., 2015). Jitkritsadakul et al. (2017) reported the tremor glove to be a medical device that incorporates a tremor detection module and electrical muscle stimulation (EMS) to suppress resting hand tremors. This device included an adjustable glove with embedded inertial sensors and an EMS module, a control box that can be attached to the belt, and a smartphone with the device’s application installed. The glove was worn on the most affected hand. Results showed that the tremor glove effectively suppressed intractable resting hand tremor among patients with PD with no serious adverse events.

The MOTIMOVE system (3F-Fit Fabricando Faber, Serbia) has multiple stimulators that can selectively activate antagonistic muscles in the forearms. This system delivers an out-of-phase stimulation. A pilot study of MOTIMOVE showed 67% tremor suppression in patients with ET and PD (Popovic Maneski et al., 2011). The TREMOR neurorobot is similar to the MOTIMOVE system and comprises electrodes to provide stimulation, sensors to evaluate biomechanical signals of tremor, and a controller. The device uses co-contraction methods that apply continuous stimulation to antagonistic muscles to increase limb stiffness and reportedly showed a 52% tremor suppression (Gallego et al., 2013).

Orthostatic tremor is characterized by unsteadiness in standing and improvement when sitting or walking (Lee et al., 2011), and has been reported in elderly patients with PD. Primary orthostatic tremor (POT) is a rare disorder characterized by 13–18 Hz tremors in the legs when standing and is often refractory to medical treatment. Lamy et al. (2021) investigated the potential beneficial effects of trans-spinal direct current stimulation (tsDCS) in POT. Their results showed that cathodal-tsDCS reduced both tremor amplitude and frequency and lowered corticospinal excitability, whereas anodal-tsDCS reduced tremor frequency only. A single session of tsDCS significantly improved POT-induced instability; thus, Lamy et al. (2021) hypothesized that tsDCS may induce spinal and supraspinal effects via ascending pathways.

##### Passive Devices

Contrastingly, passive devices absorb vibration energy by damping (Table 2). A passive device using a viscous beam and a hand orthosis using air dashpots have been reported (Kotovsky and Rosen, 1998; Takanokura et al., 2011). Buki et al. (2018) reported on a passive absorber, dubbed the vib-bracelet, a tremor attenuation device that was designed to operate as a dynamic vibration absorber. The vib-bracelet does not include any motors or sensors and is relatively light and compact. Meanwhile, the Liftware Steady™ (Liftware, United States) has an electronic stabilizing handle and a variety of attachments such as a spoon, fork, and spork, allowing patients with tremors to eat more easily. The handle contains sensors to detect motion and an onboard computer to differentiate between involuntary and voluntary movements. Sabari et al. (2019) compared the Liftware Steady™, standard spoon, weighted spoon (with standard handle and built-up handle), and swivel spoon. Reportedly, participants tended to select either a weighted spoon with a standard handle or the Liftware Steady™. A positive change in the Fahn-Tolosa-Marin Tremor Rating Scale, indicating improvement in tremors, has been shown in a pilot study (Sabari et al., 2019).

#### Non-invasive Devices for Gait Impairments

Patients with PD manifest gait disturbances and falls, which cause a significant reduction in their quality of life. The gait of PD patients is characterized by decreased step length, angular displacement, the velocity of the lower and upper limbs, high variability in step timing, poor bilateral coordination, and asymmetrical leg function (Lewis et al., 2003). Patients with PD also have a common and paroxysmal symptom termed FOG. FOG is defined as “an episodic inability (lasting seconds) to generate effective stepping in the absence of any known cause other than parkinsonism or high-level gait disorders” (Giladi and Nieuwboer, 2008). Generally, the initiation of walking requires coordination between locomotion, postural stability, and sensory-motor integration (Hass et al., 2005; Mille et al., 2012). Patients with PD have impaired kinesthesia due to degeneration of this system, resulting in inadequate motor planning to initiate movement, which causes FOG (Konczak et al., 2009). FOG is considered to be a motor manifestation of PD and appears in a significant number of patients as the disease progresses (Giladi and Nieuwboer, 2008), affecting up to 81% of patients after 20 years of disease progression (Hely et al., 2008). It is also one of the main causes of difficulty in walking and deteriorating quality of life (Moore et al., 2007; Perez-Lloret et al., 2014; Walton et al., 2015). Additionally, FOG can increase the risk of falls and cause fractures (Riancho-Zarrabeitia and Delgado-Alvarado, 2017), making freeze control an important issue. The mechanisms underlying FOG are still highly debated. However, impaired sensory processing primarily arising from the proprioceptive system is speculated to be important (Tan et al., 2011). Unfortunately, current pharmacological or surgical treatment has limited efficacy for FOG. Bilateral DBS of the STN may improve FOG during the off-period (Krack et al., 2003) but may be insufficient for long-term benefits (Kim et al., 2019) and FOG in the on-period (Davis et al., 2006). Therefore, alternative non-pharmacological/non-surgical approaches are being explored in an attempt to improve FOG. This section hereafter describes the specific methods for improving gait and FOG.

##### Non-invasive Brain Stimulation

Among several NIBS techniques, repetitive transcranial magnetic stimulation (rTMS) is particularly effective in modulating corticospinal excitability (Pascual-Leone et al., 1994; Zanjani et al., 2015). rTMS affects cortical networks both during and 1 h after stimulation (Hallett et al., 1999). rTMS can both modify the excitability of local interneurons (local effects) and induce changes in the excitability of spatially distant, but functionally interconnected, cortical areas (network effects) (Hallett et al., 2000).

High-frequency rTMS stimulation (> 5 Hz), applied to the primary motor cortex (M1), efficiently improved the motor symptoms of PD subjects (Khedr et al., 2003; Lefaucheur et al., 2004; Elahi et al., 2009; Zanjani et al., 2015). Specifically, gait performance has been improved by stimulating the leg region of the motor cortex (Maruo et al., 2013; Lee et al., 2014; Kim et al., 2015; Yokoe et al., 2018). In addition to the M1, the supplementary motor area (SMA) and pre-SMA are also used as targets for stimulation. Accumulating evidence suggests that the SMA plays a pivotal role in the pathogenesis of FOG (Snijders et al., 2011; Shine et al., 2013; Peterson et al., 2014). Several studies have explored the clinical efficacy of high-frequency rTMS over the SMA on FOG in patients with PD (Lee et al., 2014; Mi et al., 2019; Mi et al., 2020). Lee et al. (2014) explored the therapeutic effect of rTMS on the SMA and M1. In the study, the number of freezing episodes was significantly decreased after SMA stimulation, and there was a trend for a greater reduction in freezing episodes with SMA stimulation than M1 stimulation. Mi et al. (2019) investigated the effect of rTMS on the SMA with a 4-week follow-up and found significant improvements and interaction effects in the Freezing of Gait Questionnaire score and UPDRS Part III. The pre-SMA is one of the major regions that connect to the basal ganglia and prefrontal cortex and is implicated in the regulation of emotion, motor function, and behavior. A resting-state functional magnetic resonance imaging study indicated that high-frequency rTMS over the SMA confers a beneficial effect jointly by normalizing abnormal brain functional connectivity patterns specifically associated with FOG in addition to normalizing overall disrupted connectivity patterns seen in PD (Mi et al., 2020).

Eye movement disorders have been documented in PD subjects, especially in patients with FOG (Terao et al., 2013). Saccades are controlled by various brain regions and different patterns of saccade impairment reflect pathologies in the corresponding brain regions (Anderson and MacAskill, 2013). Patients with FOG show significantly worse anti-saccade performance, indicating mutually impaired inhibitory control for gait and anti-saccade (Ewenczyk et al., 2017). Okada et al. (2021) confirmed that bilateral 10 Hz rTMS to the leg region of the MC improved MDS-UPDRS motor scores and the anti-saccade success rate, both of which require adequate inhibition of the reflexive response. The improvement in the anti-saccade success rate was correlated with that of the postural instability gait difficulty sub-scores of the MDS-UPDRS (Okada et al., 2021).

Repetitive trans-spinal magnetic stimulation (rTSMS) has been confirmed to improve camptocormia, a treatment-resistant postural abnormality observed in patients with PD. Arii et al. (2014) compared rTSMS (a train of 40 stimuli) groups to sham stimulation groups in patients with PD subjects with camptocormia. The flexion angle in the standing position significantly decreased by a mean of 10.9° after rTSMS but showed no change after sham stimulation. The flexion angle while sitting significantly decreased by 8.1° after rTSMS, whereas the sham treatment had no significant effect. The authors speculated that the effect of rTSMS was possibly due to a blockade of afferent sensory nerve fibers or disruption of akinetic corticostriatal activity.

Transcranial direct current stimulation (tDCS) is a NIBS method that modulates cortical activity. tDCS induces synaptic plasticity changes after stimulation, which has lasting effects (Nitsche and Paulus, 2000) and has some advantages over rTMS. tDCS is less expensive, provides a reliable sham stimulation condition, may lead to longer-lasting modulatory effects of cortical function, and is easy to administer and perform. In animal models, tDCS increased extracellular striatal dopamine levels (Tanaka et al., 2013). Recent systematic reviews have confirmed that tDCS improves motor function in patients with PD (Ferrucci et al., 2016; Lee et al., 2019; Beretta et al., 2020a). In these studies, tDCS protocols primarily targeted motor and prefrontal cortices (e.g., the M1 and dorsolateral prefrontal cortex [DLPFC]) since brain activation patterns in these brain regions are highly involved in successful locomotion performance in patients with PD. Anodal tDCS to the M1 has been shown to significantly improve motor performance and gait in patients with PD. A significant improvement in gait with a reduction in the number and duration of FOG episodes, along with a significant reduction in the UPDRS Part III score, was observed after anodal stimulation (Valentino et al., 2014). Thus, tDCS could potentially be used in walking training and has the potential to enhance its benefits (Kaski et al., 2014b; Schabrun et al., 2016; Costa-Ribeiro et al., 2017; Yotnuengnit et al., 2018). Some studies have shown that tDCS, in addition to gait training, did not produce clinically important effects on gait speed, stride length, or cadence in people with mild to moderate disabilities associated with PD (Nascimento et al., 2021).

The DLPFC plays an essential role in dual-tasking that requires flexibility between the two tasks performed simultaneously (Collette et al., 2005). Therefore, tDCS stimulation of the DLPFC mainly affects cognitive function. Bueno et al. (2019) showed improvements in verbal fluency and reaction time on the Stroop test. A systematic review confirmed that certain cognitive deficits in PD were improved by anterior cranial tDCS (Dinkelbach et al., 2017; Putzolu et al., 2018; Mishra and Thrasher, 2021). Multitarget stimulation of motor (M1) and cognitive (left DLPFC) networks showed greater improvement than M1 stimulation only or sham stimulation in both the Timed Up and Go (TUG) and the Stroop tests (Dagan et al., 2018).

Photobiomodulation (PBM) is also being considered for use in PD therapy. PBM therapy uses a narrow wavelength band of non-thermal light (LED or laser) to modulate the cellular response and it acts at the cellular and mitochondrial levels. In recent years, transcranial PBM therapy for mental disorders such as depression and neurodegenerative diseases such as Alzheimer’s disease and PD has been attracting attention (Hamblin, 2016). Liebert et al. (2021) conducted an RCT to evaluate the efficacy of PBM in reducing the clinical signs of PD. In this study, 12 participants with idiopathic PD underwent PBM therapy for up to 52 weeks. The primary outcome measure was mobility assessment using TUG which showed significant improvements. Moreover, cognition, dynamic balance, and fine motor skills were significantly improved.

As aforementioned, various effects have been reported for each NIBS device. Especially for FOG and postural abnormality, treatment with invasive devices such as DBS is less effective, therefore it is expected to be an alternative or adjunctive treatment for patients with these conditions. However, relatively few studies have been conducted on severely injured patients, which may limit the interpretation of the results.

##### Cueing Device for Freezing of Gait

Cueing with external stimuli has also been used to assist patients with PD with FOG. Various effects have been reported, and the European guidelines on PD strongly recommend the use of cues to improve walking speed (Keus et al., 2014). Studies investigating the effect of cues on the disease stage show that the effect of cues can be obtained from the early stages of PD and increases as the disease progresses (Lirani-Silva et al., 2019). External stimulus cues for improving FOG are mainly classified into spatial information that informs the user where to guide the action (e.g., floor lines) and temporal cues that provide information about the timing of movement (e.g., metronome, vibration stimuli). An example of a visual cue is the horizontal line on the floor, which has been shown to improve gait, stride length, and the beginning of walking (Lewis et al., 2000; Jiang and Norman, 2006; Lim et al., 2006). In addition, laser visual cues are often used to improve gait performance, such as stride length and walking speed (Donovan et al., 2011; Buated et al., 2012; Ferraye et al., 2016; Tang et al., 2019). Some laser cues have been developed with lasers built into the cane (Buated et al., 2012) or attached to shoes (Ferraye et al., 2016). Another study comparing the effect of a lateral line on the floor and a wearable laser light showed that the step length increased under both conditions, but the improvement in the sequence effect—which refers to the gradual decrease in amplitude—only occurs with a lateral line on the floor (Cao et al., 2020). The presentation of cues using smart glasses has also been considered as an application of this emerging technology (Zhao et al., 2016; Janssen et al., 2017), but concerns have been expressed about the specifications of smart glasses, such as weight and the reduction of the field of view due to the frame (Janssen et al., 2017).

Some studies have also shown that auditory cues can improve FOG (Delval et al., 2014; Lopez et al., 2014; Ginis et al., 2017a; Spildooren et al., 2017). Patients with PD release their anticipatory postural adjustments more quickly when auditory cueing is applied. Monotonous external inputs, such as a metronome, are often used with/without other non-auditory cues (Bachlin et al., 2010; Espay et al., 2010; Hove et al., 2012; Baram et al., 2016; Ginis et al., 2016, 2017b; Zhao et al., 2016). Hove et al. (2012) used a non-linear limit-cycle oscillator to interactively generate a rhythmic pacing sequence using the information on the number of steps taken from a shoe-mounted foot pressure sensor to improve walking.

However, some side effects of these cueing devices have also been identified. People with PD demonstrated cue dependency, expressed as a decline in movement after cue removal (Nieuwboer et al., 2009; Spildooren et al., 2012; Vercruysse et al., 2012). It has also been suggested that external cues may have a negative effect on increasing gait variability because they increase the cognitive load, as all steps need to be coordinated to synchronize (Yogev et al., 2005). These characteristics limit the effectiveness of simple cueing. To apply appropriate cueing at the required moment, effective FOG detection technologies are warranted. Ginis et al. (2017b) proposed an intelligent system in which an inertial sensor attached to the foot was used to obtain the average cadence, and auditory cues and auditory feedback were triggered only when the cadence deviated from the reference by more than 5%. This method significantly reduced cadence deviation compared to the use of normal cues. Additionally, Bachlin et al. (2010) developed a wearable system that initiated auditory cueing only when FOG was detected. Moreover, some studies have shown self-generating internal cues while singing a song instead of providing cues successfully improves gait externally (Harrison et al., 2019). The effect of internal cueing, in the form of singing, vs. external cueing, in the form of listening to music, gait was examined in people with and without PD. Internal cueing significantly improved cadence compared to external cueing (Harrison et al., 2018), and external cueing increased gait variability whereas internal cues did not (Harrison et al., 2019; Horin et al., 2020). Since higher gait variability is associated with falls, this finding is important for stable walking when addressing PD with FOG.

##### Non-invasive Vagus Nerve Stimulation

Invasive vagus nerve stimulation (VNS), delivered by a surgically implanted device, has been approved as an adjunctive neuromodulation therapy for epilepsy (Terry, 2014). VNS is supposed to affect various brain regions through direct effects on the nucleus tractus solitarius and locus coeruleus (Kraus et al., 2007; Oshinsky et al., 2014). Anti-inflammatory properties of VNS have been suggested (Corcoran et al., 2005; Majoie et al., 2011) and potential applications to a wide range of inflammatory disorders have been advocated (Bonaz et al., 2016). Neuroinflammation has been implicated in the pathogenesis of PD (Akiyama et al., 2000); therefore, VNS may be effective in the treatment of PD.

Recently, the development of portable, non-invasive VNS (nVNS) devices has simplified this treatment modality (Yuan and Silberstein, 2016). Notably, the application of nVNS in patients with PD has shown beneficial effects for FOG (Mondal et al., 2019). In the study, the application of 2 × 120 s acute nVNS improved the number of steps taken while turning and reduced UPDRS Part III scores in patients with FOG induced by PD by two-dimensional spatiotemporal gait parameter analysis. These short-term effects are most likely due to indirect activation of central neural circuits, including noradrenergic projections from the locus coeruleus (Johnson and Wilson, 2018), a brain region implicated in the pathogenesis of FOG (Ono et al., 2016). Morris et al. (2019) found significant improvement in step length variability in patients with PD after a single application of cervical nVNS.

A randomized, double-blind, sham-controlled crossover study in which nVNS was applied three times a day for 1 month to confirm the long-term effects of cervical nVNS (Mondal et al., 2021), showed improvement in motor function in patients with PD and a significant reduction in serum inflammatory markers. Intriguingly, the analysis of serum biomarkers after nVNS showed a reduction in neuroinflammatory markers (Tumor Necrosis Factor-α) along with increased brain-derived neurotrophic factor (BDNF), which implicated increased neuroplasticity. Given that neuroinflammation has been implicated in the pathogenesis of PD (Akiyama et al., 2000), nVNS may have disease-modifying effects.

##### Vibrotactile Stimulation Devices

Vibration stimuli to the muscles act as a powerful proprioceptive input and strongly affect the motion perception during extremity movements of healthy people and patients with various neurological disorders (Cohen and Starr, 1985; Han et al., 2014). Peripheral vibrotactile stimulation has been shown to improve bradykinesia, FOG, gait impairment, and postural instability in patients with PD, possibly by affecting the central pattern generator in a desynchronized state (Zehr and Haridas, 2003; Volpe et al., 2014; Pereira et al., 2016; Syrkin-Nikolau et al., 2018; Peppe et al., 2019; Serio et al., 2019; Spolaor et al., 2021; Tan et al., 2021). Three possible mechanisms for how vibration stimuli work against FOG in patients with PD have been laid forth: (i) a cueing effect, (ii) modulation effect against impairment in dealing with conflicting cognitive/attentional resources, and (iii) enhanced proprioceptive processing (Pereira et al., 2016).

Peripheral vibrotactile stimulation delivered by a C-2 tactor glove (Engineering Acoustics Inc., Florida, United States) with high-frequency trains in a patterned sequence acutely improved gait asymmetry, arrhythmicity, and wrist bradykinesia in patients with PD (Syrkin-Nikolau et al., 2018). The study indicated that peripheral vibrotactile stimulation is safe and its effect continues even 1 month after stimulation. The use of light-touch contact is being considered as another approach. Light-touch contact is a phenomenon in which a part of the body lightly (<1 N) touches a stable surface to obtain sensory feedback about the orientation of the body and limbs in space, which can activate the postural muscles and adjust the axial tension of the body’s posture. Light-touch contact has also been found to attenuate postural sway in patients with PD, although this is a commonly occurring effect (Franzen et al., 2012; Rabin et al., 2013). Walking has been found to improve the center of gravity shift and improve gait initiation (Ditthaphongphakdee and Gaogasigam, 2021). A wearable device using vibration stimuli on the fingertips provides tactile feedback reportedly improved body balance in normal subjects (Shima et al., 2021) and may also be effective for PD subjects.

Although the improvement rates were small, muscle vibration therapy in the lower limbs using eight vibrators (60 Hz) made by the study authors, significantly improved stride length and walking speed in patients with PD (Han et al., 2014). Another study reported that hand-made vibratory devices embedded in elastic insoles (70 Hz), where the vibratory device senses the pressure on the sole and delivers the vibration stimulus during walking, decreased stride variability while increasing walking speed, stride duration, stride length, and cadence in patients with PD (Novak and Novak, 2006). Admittedly, these changes were significant but small. Another study placed the CUE1 (Charco Neurotech Ltd., London, United Kingdom) on the patient’s sternum to produce focused vibrotactile pulsatile stimuli (Tan et al., 2021). Since this device was effective for FOG when it was turned on before the FOG episodes, the authors hypothesized that CUE1 has a cueing effect in addition to enhanced proprioceptive stimuli. Another study revealed that focal vibration training using Equistasi^®^ (Equistasi^®^, Italy) improved postural stability in patients with PD. The Equistasi^®^ is composed of nanotechnological particles that transform the body temperature into a mechanical high-frequency vibration (0.8 N, 9000 Hz). The device is attached over the seventh cervical vertebra and on each soleus muscle-tendon to alleviate gait disturbances and postural instability (Volpe et al., 2014; Peppe et al., 2019; Serio et al., 2019; Spolaor et al., 2021). RCTs using Equistasi^®^ showed significantly improved motor performance in patients with PD.

Another study revealed that a custom-made vibration device (100 Hz and 1.2 mm of amplitude) attached to the less affected limb moderately alleviated FOG in patients with PD when vibration is applied following FOG onset (Pereira et al., 2016). Importantly, no positive effects for FOG were identified when the vibration device was applied before the FOG onset, which indicated that vibration did not serve as an external cue in the study. Additionally, vibration stimuli did not exert a positive effect when the more affected limb triggered attentional processing by vibration, indicating that the modulation effect of choosing conflicting cognitive/attentional resources is unlike the mechanism underpinning the effect (Pereira et al., 2016). Given that improper sensory feedback causes worsening of movements (Nieuwboer et al., 2009), vibration stimuli in the lower extremities strongly enhance proprioceptive information and play an important role in controlling posture and balance (Sorensen et al., 2002; Pereira et al., 2016), and the more preserved side of the basal ganglia seems to properly process the enhanced proprioceptive information (Pereira et al., 2016). Upper limb freezing in PD patients with FOG is associated with decreased activation in the basal ganglia and increased activation in the frontal lobes, including the supplementary and primary motor cortices, which indicated the impairment in the indirect pathway-driven non-selective inhibitory program through the cortico-basal ganglia-thalamo-cortical feedback loop in FOG pathogenesis (Vercruysse et al., 2014). Since local vibration stimuli are reported to evoke focused activation of motor cortical circuits (Rosenkranz and Rothwell, 2003), they may help focus on the desired movements through the thalamus.

##### Mechanical/Electrical Peripheral Stimulation Devices

Peripheral sensory deficits, particularly reduced plantar sensitivity, have been reported in PD (Pratorius et al., 2003; Nolano et al., 2008). This reduction may result from the loss of cutaneous receptors, encapsulated endings, or free nerve endings (Nolano et al., 2008), and is associated with reduced control of compensatory stepping and impaired balance, which causes falls (Pratorius et al., 2003; Kerr et al., 2010).

Textured insoles significantly decreased mediolateral postural sway, which indicated an improvement in standing balance (Qiu et al., 2013). The continuous use of textured insoles for 1-week increased stride length, and the effect continued for another week without textured insoles (Lirani-Silva et al., 2017). Ribbed insoles produce a significant increase in single-limb support time and normalization of the muscle activation sequence of the tibialis anterior in patients with PD (Jenkins et al., 2009). As described in the previous section, vibration stimulation at the soles increased walking speed and improved stride variability in PD subjects (Novak and Novak, 2006). However, the improvement rates in these studies were usually under 20%.

The automated mechanical peripheral stimulation (AMPS) device Gondola™ (Gondola Medical Technologies SA, Switzerland) has been proven effective for addressing motor impairment in patients with PD. Immediate and long-term increases in stride length and gait speed with Gondola™ have been reported (Kleiner et al., 2015; Stocchi et al., 2015). The benefit was maintained for 10 days after the last treatment (Stocchi et al., 2015). Based on these studies, a randomized controlled clinical trial using Gondola™ was performed involving patients with PD, and Gondola™ promoted faster walking with longer strides after 6–8 stimulation sessions (Galli et al., 2018; Kleiner et al., 2018; Pagnussat et al., 2018, 2020; Pinto et al., 2018). Contrastingly, there was no positive effect in terms of improving static postural control in individuals with PD and FOG (Prusch et al., 2018). Interestingly, patients with PD who underwent treatment with Gondola™ showed higher serum BDNF and lower serum cortisol levels in accordance with improvements in gait parameters (Pagnussat et al., 2018). Gondola™ did increase gait velocity and resting-state brain connectivity between the basal ganglia and sensory-related brain areas (insular and somatosensory cortices) in PD subjects. Gait velocity is positively correlated with increased connectivity between the sensory, motor, and supplementary motor cortices (Pagnussat et al., 2020). These findings may help elucidate the mechanism by which AMPS influences gait.

The electrical stimulation of the common peroneal nerve immediately improved average stride length during a three-min walk and reduced the frequency of falls and FOG during an 8-week stimulation period which diminished 4 weeks after the cessation (Mann et al., 2008). A single randomized controlled trial (RCT) showed significant improvement in 10 m walking speed at week 18 compared to the standard care group (Taylor et al., 2021). However, the decrease in the UPDRS score was not significant and the improvement in 10 m walking speed disappeared 4 weeks following the stimulation session.

To reduce FOG, the use of a “fixed” rhythmic sensory electrical stimulation (sES) cueing strategy has been proposed (Rosenthal et al., 2018). sES cueing was delivered by a voltage-controlled two-channel stimulator and skin surface electrodes were placed over the hamstring or quadriceps muscle. In this study, nine patients with PD walked while receiving sES cueing and their performance was evaluated. Results showed that sES cueing significantly reduces the time taken for the walking task and in the number of FOG episodes by 14.23 and 58.28, respectively.

### Systematic Review

For the systematic review, we identified RCT studies in the last 10 years (2011–2021). Concerning the invasive devices, RCTs for DBS and LCIG were extracted. These devices have already received FDA certification and a CE mark and are currently being used. The results show a higher efficacy than non-invasive devices. There were no RCTs on SCS and continuous subcutaneous infusion of levodopa.

As represented in Table 1, most RCTs refer to non-invasive devices. RCTs for tremors and cueing devices were few despite many devices having been developed. In contrast, extensive RCTs of rTMS and tDCS for various targets and frequencies have been performed. Most of the rTMS studies showed improvement in motor function as assessed by TUG and UPDRS Part III. rTMS is FDA approved for obsessive-compulsive disorder and depression but has not yet been approved for PD. Several studies have targeted M1 for the improvement of motor function, and most of them have shown that it improves motor function. The second most common target was SMA; some studies showed improvement in FOG as well as improvement in motor function. tDCS has also been used to target M1 and DLPFC. tDCS over M1 has been reported to improve motor function. tDCS over DLPFC may not directly improve motor function, but may improve performance under certain conditions, such as dual-tasking. The effects of tDCS, when used in combination with physical training, have also been studied extensively. There are multiple reports of improved gait performance when stimulated in conjunction with walking. Mechanical/electrical peripheral stimulation devices have also been the subject of many RCTs, which have reported effectiveness in gait performance. Most of the studies have been with Gondola™, a device that is FDA certificated and CE marked. As shown in Table 1, RCTs have been conducted on other non-invasive devices and have shown improvement in motor symptoms, but the number of studies is small, and certification has not been obtained. There is a big difference between invasive and non-invasive treatments in terms of treatment effectiveness. For example, when comparing the improvement of UPDRS Part III, DBS may have an effect of more than 15 points, while non-invasive devices usually have an effect of a few points, and none of them improve by more than 10 points.

## Discussion

This paper describes the progress made in the development of therapeutic devices for motor symptoms in patients with PD. We have conducted a narrative literature review and a systematic review of recent RCTs. Generally, invasive devices are more effective than non-invasive devices in terms of improving motor function in patients with PD. DBS has a striking effect on motor symptoms in PD subjects and, specifically, aDBS is more effective than cDBS, pointing to the importance of instrumental advances. Little et al. (2013) confirmed that motor scores improved during aDBS were 27% better than cDBS. In the future, it is expected that a closed-loop system in which the release of neurotransmitters is a control variable will be realized by FSCV and that a more accurate DBS system might be developed. SCS with burst waveform stimulation showed acute and larger improvements in both motor symptoms, including tremor and gait parameters (Kobayashi et al., 2018; Furusawa et al., 2020). Continuous infusion of levodopa-carbidopa via an intestinal infusion (Duodopa^®^) system has also demonstrated significantly decreased off-time and increased on-time without troublesome dyskinesia (Olanow et al., 2014). In recent years, as a less invasive method, the continuous subcutaneous infusion of levodopa or apomorphine using ND0612 or ND0701 was also developed (Ramot et al., 2018; Olanow et al., 2021; Larson and Simuni, 2022). The effects of these invasive medical devices are usually drastic.

Regarding non-invasive medical devices, we introduced several devices that are effective for tremors, gait, and FOG. These devices have seen improved efficacy and have the advantage of being non-surgical and easy to use. Thus, each device has its characteristics and effects, and the proper selection and use of these devices will result in more effective treatment. As shown in the systematic review, a growing number of high-evidence studies of non-invasive devices have been conducted in recent years. In particular, rTMS has been subjected to meta-analysis and is more reliable than other devices (Elahi et al., 2009; Zanjani et al., 2015). The meta-analysis revealed that targeting M1 improved UPDRS and that targeting multiple brain regions had a short-term effect on functional locomotion. In addition, improvement in gait was reported when combined with physical training (Kaski et al., 2014b; Costa-Ribeiro et al., 2017; Yotnuengnit et al., 2018; Lee and Kim, 2021). rTMS and tDCS have been studied for different stimulation targets, and different effects were reported for each. A relatively large number of RCTs implies that researchers who performed rTMS and tDCS have some confidence in their efficacy on motor symptoms in patients with PD. Further research is required to determine the best treatment method and target. Significant effects have also been reported for other non-invasive devices, such as nVNS, vibrotactile stimulation devices, mechanical peripheral stimulation, and sensory electrical stimulation by RCT. Non-invasive devices are less effective than invasive devices, but easy-to-use and less invasive characteristics make their use more feasible and easier. They may grow more prevalent in the upcoming decade. They also have the potential to become standard treatments for PD in the next generation.

The devices introduced in this paper include prototypes, which need to be further developed for practical use. Since it is important to develop safe and reliable products, it is very important to make effective, reliable, and easy-to-use gold-standard criteria to evaluate them.

## Author Contributions

JF, RM, and NY designed and drafted the manuscript. All authors revised and approved the final version of the manuscript.

## Conflict of Interest

The authors declare that the research was conducted in the absence of any commercial or financial relationships that could be construed as a potential conflict of interest.

## Publisher’s Note

All claims expressed in this article are solely those of the authors and do not necessarily represent those of their affiliated organizations, or those of the publisher, the editors and the reviewers. Any product that may be evaluated in this article, or claim that may be made by its manufacturer, is not guaranteed or endorsed by the publisher.

## Abbreviations

aDBS: adaptive deep brain stimulation
AMPS: automated mechanical peripheral stimulation
BDNF: brain-derived neurotrophic factor
cDBS: conventional deep brain stimulation
DBS: deep brain stimulation
DLPFC: dorsolateral prefrontal cortex
EEG: electroencephalography
EMG: electromyography
EMS: electrical muscle stimulation
ET: essential tremor
FDA: Food and Drug Administration
fMRI: functional magnetic resonance imaging
FTM-TRS: Fahn-Tolosa-Marin Tremor Rating Scale
GPi: internal globus pallidus
FOG: freezing of gait
FOGQ: Freezing of Gait Questionnaire score
FSCV: fast scan cyclovoltammetry
LCIG: continuous infusion of levodopa-carbidopa gel
LFPs: local field potentials
M1: primary motor cortex
NIBS: non-invasive brain stimulation
nVNS: non-invasive vagus nerve stimulation
PBM: photobiomodulation
PD: Parkinson’s disease
POT: primary orthostatic tremor
RCT: randomized controlled trial
rTMS: repetitive transcranial magnetic stimulation
PMC: premotor cortex
RMS: root mean square
ROM: range of motion
rTSMS: repetitive trans-spinal magnetic stimulation
SCS: spinal cord stimulation
SEMG: surface electromyography
sES: sensory electrical stimulation
SMA: supplementary motor area
STN: subthalamic nucleus
tDCS: transcranial direct current stimulation
TEEDs: total electrical energy delivered to the tissues per second
TETRAS: the Tremor Research Group’s Essential Tremor Rating Assessment Scale
tsDCS: trans-spinal direct current stimulation
TUG: timed up and go
UPDRS: the Unified Parkinson’s Disease Rating Scale
VIM: ventral intermediate nucleus of the thalamus
VNS: vagus nerve stimulation.
